# Chemical Composition and *In Vitro* Cytotoxic Activity of Essential Oil of Leaves of *Malus domestica* Growing in Western Himalaya (India)

**DOI:** 10.1155/2012/649727

**Published:** 2012-04-29

**Authors:** Mayanka Walia, Tavleen S. Mann, Dharmesh Kumar, Vijai K. Agnihotri, Bikram Singh

**Affiliations:** ^1^Natural Plant Products Division, CSIR-Institute of Himalayan Bioresource Technology, P.O. Box No. 6, Palampur 176 061 (HP), India; ^2^Biotechnology Division, CSIR-Institute of Himalayan Bioresource Technology, P.O. Box No. 6, Palampur 176 061 (HP), India

## Abstract

Light pale-colored volatile oil was obtained from fresh leaves of *Malus domestica* tree, growing in Dhauladhar range of Himalaya (Himachal Pradesh, India), with characteristic eucalyptol dominant fragrance. The oil was found to be a complex mixture of mono-, sesqui-, di-terpenes, phenolics, and aliphatic hydrocarbons. Seventeen compounds accounting for nearly 95.3% of the oil were characterized with the help of capillary GC, GC-MS, and NMR. Major compounds of the oil were characterized as eucalyptol (43.7%), phytol (11.5%), *α*-farnesene (9.6%), and pentacosane (7.6%). Cytotoxicity of essential oil of leaves of *M. domestica* was evaluated by sulforhodamine B (SRB) assays. The essential oil of leaves of *M. domestica*, tested against three cancer cell lines, namely, C-6 (glioma cells), A549 (human lung carcinoma), CHOK1 (Chinese hamster ovary cells), and THP-1 (human acute monocytic leukemia cell). The highest activity showed by essential oil on C-6 cell lines (98.2%) at concentration of 2000 *μ*g/ml compared to control. It is the first paper in literature to exploit the chemical composition and cytotoxic activity of leaves essential oil of *M. domestica*.

## 1. Introduction

*Malus domestica* (Rosaceae), a small and deciduous tree, well known by the whole world for its delicious and nutritious fruits which are being grown in temperate region of the world [1]. Its leaves are between 5–12 cm long and 3–6 cm wide. It blossoms in the spring and has white flowers with a pink tint. About 25 to 47 different *Malus* species are known, among which around seven species are taxonomically close to *M. domestica* [2, 3]. Indian Himalayas cover an approximately 14% of the total geographical area [4] with apple as the main cash crop. In India, cultivation of apple is mainly restricted to Himalayan regions of Jammu and Kashmir, Himachal Pradesh and Uttaranchal, comprising of several varieties [5].

*M. domestica* exhibit efficient antioxidant property owing to the presence of its phytoconstituents. These phytoconstituents are also well known to have anti-inflammatory, antiviral and antimicrobial properties [6, 7]. Apple fruit is considered nutritious for human health as it has health-beneficial constituents such as dietary fibre, sugars, vitamins, and phenolic compounds [8], which are responsible for curing cancer, cardiovascular disease, asthma, and diabetes [9]. Malic acid is the predominant organic acid in apple fruits, and it helps to maintain the liver in a healthy condition also help in digestion process [1]. The essential oil composition of its flowers and fruits has been reported earlier [10–12].

This work is in continuation of the screening programme and chemical investigation of unexploited flora of Western Himalaya for new sources of essential oils and aroma chemicals. In this paper, we have carried out qualitative and quantitative study of extracted essential oil and its cytotoxicity against carcinoma cell lines.

## 2. Materials and Methods

### 2.1. Plant Material

Fresh *M. domestica* leaves (Variety Golden) were collected from Chindi village of Mandi district situated at an elevation of 3300 m above sea level located in the mid-hills of the Western Himalayas (Dhauladhar range) during May-June 2011. The plants were authenticated by Dr. Brij Lal, taxonomist at the institute and voucher specimen deposited in herbarium of the IHBT, Palampur, India (voucher no. PLP 11688).

### 2.2. Hydrodistillation

Two kilograms of fresh leaves of *M. domestica* were hydrodistilled to obtain essential oils by using Cleavenger-type apparatus for three hours. Each sample afforded pale-colored oil with eucalyptol as a dominant constituent with characteristics fragrance (yield 0.004%; moisture-free basis 0.01%). The oils were dried over anhydrous sodium sulphate and placed at low temperature until used for further analysis. The whole oil was examined with the help of ^1^H NMR (300 MHz) and ^13^C NMR (75.4 MHz), run on Bruker Avance 300 MHz spectrometer in deuterated chloroform solution with TMS as an internal standard.

### 2.3. GC Analysis and Quantification

The composition of the oil was carried out by GC on Shimadzu GC 2010 equipped with DB-5 (J&W Scientific, Folsom, CA, USA) fused silica capillary column (30 m ×0.25 mm i.d.; 0.25 *μ*m film thickness) and FID. The GC oven temperature programme was as follows: 90°C (initial temperature) held for 2 minutes and then at a rate of 7°C/min to 220°C and held for 5 minutes. “Injector temperature, 240°C”; “detector temperature, 260°C”; injection mode, split. Carrier gas was helium at column flow rate of 1.05 mL/min (100 kPa).

 Retention indices (RI) of the sample components and authentic compounds were determined on the basis of homologous *n*-alkane hydrocarbons under the same conditions. The quantitative composition was obtained by peak area normalization, and the response factor for each component was considered to equal 1.

### 2.4. GC/MS Analysis and Identification

The GC/MS analyses were conducted using a Shimadzu QP 2010 using a DB-5 (J&W Scientific, Folsom, CA, USA) capillary column (30 m × 0.25 mm i.d.; 0.25 *μ*m thickness). The GC oven temperature was 70°C for 3 minutes and then to 220°C at 4°C/min and held for 5 minutes. “Injector temperature, 240°C”; “Interface temperature, 250°C”; acquisition mass range, 800–50 amu; ionization energy, 70 eV. Helium was used as carrier gas. Tuning and calibration of the machine was done by Manufacturer Company.

 Compounds were identified by using library search of National Institute of Standards and Technology (NIST) database [13] as well as by comparing their mass spectral fragmentation pattern with those reported in the literature [14]. Also the identification of the oil components was carried out by comparison of their linear RI and ^13^C NMR spectra with literature [15, 16].

### 2.5. Cell Lines and Cell Culture

C-6 (glioma cells) obtained from GNDU, Amritsar. Cells were grown in DMEM (Sigma Aldrich) supplemented with 10% heat-inactivated fetal bovine serum and 1% antibiotic antimycotic (Sigma Aldrich). A549 (human lung carcinoma), cell line was obtained from Indian Institute of Integrative Medicine, Jammu. Cells were grown in RPMI-1640 (Sigma Aldrich) supplemented with 10% heat-inactivated fetal bovine serum (Sigma Aldrich) and 1% antibiotic antimycotic (Sigma Aldrich). CHOK1 (Chinese hamster ovary cells) cell line was obtained from Indian Institute of Integrative Medicine, Jammu, cells were grown in HAM'S-F-12 (Sigma Aldrich) supplemented with 10% heat-inactivated fetal bovine serum (Sigma Aldrich) and 1% antibiotic antimycotic (Sigma Aldrich). THP-1 (human acute monocytic leukemia cell) cell line was obtained from Indian Institute of Integrative Medicine, Jammu. Cells were grown in RPMI-1640 (Sigma Aldrich) supplemented with 10% heat-inactivated fetal bovine serum (Sigma Aldrich) and 1% antibiotic antimycotic (Sigma Aldrich). All the cell lines were maintained at 37°C in a 5% CO_2_ humidified atmosphere.

### 2.6. Sulphorodamine B Assay

The monolayer cell culture was trypsinized, and the cells were washed twice with phosphate-buffered saline (PBS) and incubated at a density of 4000-5000 cells/well in flat-bottomed 96-well microtiter plates in 100 *μ*L of medium with 10% fetal bovine serum (FBS) (Sigma). Several dilutions (100, 300, 1000, 1500, and 2000 *μ*g/mL against C-6, A549, and CHOK1; 100, 300, 1000 *μ*g/mL against THP-1) of the test compounds in 100 *μ*L of medium with 10% FBS were added to the wells. Mitomycin-c (1 *μ*M) was used as positive control. The plates were then incubated at 37°C for 48 hours in 5% CO_2_ incubator, microscopic examination was carried out, and observations recorded after every 24 hours. After 48 hours, 50 *μ*L of 50% trichloroacetic acid (Calbiochem) was added to the wells gently such that it forms a thin layer over the test compound, and then the plates were incubated at 4°C for one hour. After incubation period, the plates were flicked and washed five times with tap water to remove traces of medium, sample and serum, and then air-dried. Subsequently, 100 *μ*L of the SRB solution (1% in acetic acid) was added to each well of the air dried 96-well plates at room temperature. After standing for 30 minutes, the unbound dye was removed by rapidly washing five to six times with 1% acetic acid, and the plates were then air dried. 100 *μ*L of 10 mM Tris base (Sigma) was then added to the wells to solubilize the dye. The plates were shaken vigorously for 5 minutes. The absorbance was measured using microplate reader (BioTeK Synergy H1 Hybrid Reader) at wavelength of 540 nm [17]. The growth inhibition rate was calculated as percentage of parallel negative controls. The percentage growth inhibition was calculated using following formula:

(1) %  cell inhibition=100−{(At−Ab)/(Ac−Ab)}×100,

where At is the absorbance value of test compound, Ab is the absorbance value of blank, and Ac is the absorbance value of control.

### 2.7. Morphological Changes

**Figure 1:**
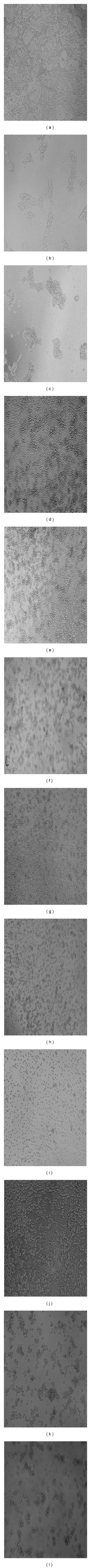
Microscopic images of cells treated with essential oil of leaves of *Malus domestica.* (a) C-6 cells without any treatment, (b) C-6 cells after 24 hrs of addition with 1000 *μ*g/mL test compound, (c) C-6 cells after 48 hrs of addition with 1000 *μ*g/mL test compound, (d) A549 cells without any treatment, (e) A549 cells after 24 hrs of addition with 2000 *μ*g/mL test compound, (f) A549 cells after 48 hrs of addition with 2000 *μ*g/mL test compound, (g) CHOK1 cells without any treatment, (h) CHOK1 cells after 24 hrs of addition with 2000 *μ*g/mL test compound, (i) CHOK1 cells after 48 hrs of addition with 2000 *μ*g/mL test compound, (j) THP-1 cells without any treatment, (k) THP-1 cells after 24 hrs of addition with 1000 *μ*g/mL test compound, and (l) THP-1 cells after 48 hrs of addition with 1000 *μ*g/mL test compound.

The morphological changes of C-6, A549, CHOK1 and THP-1 cells treated with essential oil of leaves of *M. domestica* for 24 and 48 hours were observed by using Fluorescent microscope (Nikon Eclipse T_i_) at magnification 10X (Figure 1).

### 2.8. Apoptotic Assay (Caspase-3/7 Activity)

Cells were plated in 96-well plates at 10 × 10^3^ density. After 5 hours of treatment of cells, the caspase activity detected against A549 and CHOK1 cell lines using different concentrations of essential oil (100, 300, 1000, 1500, and 2000 *μ*g/mL). The total caspase activity detected using the Apo-ONE homogeneous Caspase-3/7 assay kit (Promega). The activity was measured according to the supplier's instructions provided with the kit. The Caspase-3/7 activity was calculated by measuring the net relative fluorescence units (RFU) of respective incubated cells in the 96-well plates. The RFU was measured using microplate reader (BioTeK Synergy H1 Hybrid Reader) at an emission wavelength of 530 nm. The excitation wavelength was 485 nm. Caspase-3/7 activity is indicated by net fluorescence which is calculated by the following formula suggested by the supplier:

(2) Caspase−3/7  activity  =assay  RFU−blank  RFU.

## 3. Results and Discussions

*M. domestica* is found in the upper hill region of Himalaya (India), and it is for the first time that the essential oil obtained from leaves of *M. domestica*, collected (elevation of 3300 m above sea level) in the month of May-June 2011, is being investigated for its chemical components. The plant material collected from 10 different populations of Chindi village of Mandi district on hydrodistillation procured 0.01% light pale-colored oil on mfb with characteristic eucalyptol dominant flavour. Qualitative GC pattern of the different oil samples were found to be nearly similar (see Supplementary Material available online at doi: 10.1155/2012/649727); therefore, a representative sample of the leaves from entire area was collected and its oil composition, and biological activity was investigated.

 The oil was found to be a complex mixture of terpenes and phenolics, and a total of seventeen components were identified by RI, GC-MS and with the help of other spectroscopic methods, which accounted for 95.3% of the oil. Out of seventeen identified compounds, major seven components were accounted for nearly 83.5% of the oil composition vide GC (Table 1). The fragrance and composition of leaves essential oil was found to be widely different from the essential oil composition of fruits and flowers reported in the literature [11, 12, 18, 19] with eucalyptol (43.7%) as a dominant component. The other major components of *M. domestica* leaves essential oil were characterized as phytol (11.5%), *α*-farnesene (9.6%), pentacosane (7.6%), tricosane (4.2%), podocarpene A (3.8%), and* cis*-3-hexenyl benzoate (3.1%). The other minor components identified in essential oil were 1,6,10-dodecatrien-3-ol-3,7,11-trimethyl (2.0%), benzyl benzoate (1.7%), 2-pentadecanone (1.3%), 2-undecanone (1.1%), *trans-*caryophyllene (1.1%), 2-dodecanone (1.1%), *n*-hexyl benzoate (1.0%), linalool (1.0%), 4-terpineol (0.8%), and *β*-damascenone (0.7%). The quality of essential oils extracted from different areas of Mandi district, indicated that there is no significant qualitative variation in chemical composition, and therefore a uniform quality essential oil may be produced from leaves for its commercial production.

Numerous reports showed the high cytotoxic properties of terpenes, and phenolics against cancer cell lines [20, 21]. The effects of essential oil of leaves of *M. domestica *were tested *in vitro* against C-6 (glioma cells), A549 (human lung carcinoma), CHOK1 (Chinese hamster ovary cells) and THP-1 (human acute monocytic leukemia cell), using different concentrations (100, 300, 1000, 1500, and 2000 *μ*g/mL against C-6, A549, and CHOK1; 100, 300, and 1000 *μ*g/mL against THP-1) by sulforhodamine B (SRB) assay. The percentage of survived cells was calculated by measuring the absorbance of respective incubated cells in the 96-well plates. The results are shown in Table 2. The effect of the essential oil of leaves of *M. domestica* showed highest activity (98.2%) on C-6 (glioma cells) cell line at concentration of 2000 *μ*g/mL and comparable to the standard drug [22]. The essential oil of leaves of *M. domestica* has shown concentration-dependent activity against all the cell lines. Based on above study, significant results have been observed. The morphological changes were observed in C-6, A549, CHOK1 and THP-1 cell lines treated with essential oil of *M. domestica.* The cell number reduced with the increase in the concentration of oil. The affected cells showed some features of apoptosis such as cellular shrinkage, membrane blebbing, nuclear compaction and fragmentation, and formation of apoptotic bodies (Figure 1). Furthermore, also the mechanisms of inhibition were studied by apoptotic assay kit (Apo-ONE Homogeneous Caspase-3/7 assay kit, Promega) against A549 and CHOK1 cell lines using different concentrations (100, 300, 1000, 1500, and 2000 *μ*g/mL). Activity of Caspase-3/7 was calculated by measuring the net Relative fluorescence units (RFU) of respective incubated cells in the 96 well plate (Table 3).

## 4. Conclusions

The study on composition of essential oil of *M. domestica* leaves shows that it contains phenolics as well as other bioactive components. Eucalyptol (cyclic ether and monoterpenoid), a major constituent of oil is also well known to possess anti-microbial, anti-inflammatory properties, and has potential to kill leukaemia cells *in vitro* [23]. Phytol, (acyclic diterpenoid), the second major constituent, is usually found as a component of chlorophyll, has anti-inflammatory activity [24] and can be used as an effective anticonvulsant and antiepileptic therapeutic drug [25]. Our study on cytotoxicity of essential oil extracted from leaves of *M. domestica* against C-6, A549, CHOK1, and THP-1 cell lines shows very good pharmacological activity which suggest that this might be a potential source of anticancer activity. In the present experiment the observed anticancer activity of the oil may be accounted for synergistic effect of all the compounds present in the essential oil.

## Supplementary Material

The plant materials collected from 10 different populations of Chindi village of Mandi district on hydrodistillation procured 0.01% light pale colored oil on mfb with characteristic eucalyptol dominant flavour. All the samples were analyzed with the help of GC and GC-MS. All the samples qualitative GC pattern was found similar and the representative molecules concentration in the samples were as given below in Table 1A.

## Figures and Tables

**Table 1:** Chemical composition of *Malus domestica* essential oil from leaves.

Compounds	RI	Percentage	Method of identification
Eucalyptol	1031	43.7	MS; RI; ^13^C NMR
Linalool	1095	1.0	MS; RI; Co-GLC
4-Terpineol	1180	0.8	MS; RI; Co-GLC
2-Undecanone	1288	1.1	MS; RI
*β*-Damascenone	1379	0.7	MS; RI
2-Dodecanone	1391	1.1	MS; RI
*Trans*-Caryophyllene	1417	1.1	MS; RI; Co-GLC
*α*-Farnesene	1506	9.6	MS; RI; ^1^H NMR
1,6,10-Dodecatrien-3-ol-3,7,11-trimethyl	1542	2.0	MS; RI
*Cis*-3-Hexenyl benzoate	1567	3.1	MS; RI
*n*-Hexyl benzoate	1573	1.0	MS; RI
Benzyl benzoate	1762	1.7	MS; RI, ^1^H NMR
6,10,14-Trimethyl-2-pentadecanone	1844	1.3	MS; RI
Podocarpene A	2036	3.8	MS; RI
Phytol	2112	11.5	MS; RI; ^1^H and ^13^C NMR
Tricosane	2298	4.2	MS; RI
Pentacosane	2497	7.6	MS; RI

**Table 2:** *In vitro *cytotoxicity against different cancer cell lines by SRB assay.

Sample	Conc. (*μ*g/mL)	Growth inhibition percentage			
		C-6	A-549	CHOK1	THP-1
Essential oil of leaves of *M. domestica *	100	5.5	11.5	0	0
	300	18.8	1.5	13.5	19.9
	1000	58.5	60.7	68.3	65.7
	1500	68.0	74.5	70.8	na
	2000	98.2	76.7	69.5	na
Mitomycin C	(1 *μ*M)	65.7	76.4	75.4	na
Vinblastin	(1 *μ*M)	na	na	na	73.1

na: the activity was not done.

**Table 3:** Caspase activity against different cancer cell lines by Apo-ONE Homogeneous Caspase-3/7 assay kit (Promega).

Sample	Conc. (*μ*g/mL)	Relative fluorescence units (RFU)	
		A549	CHOK1
Essential oil of leaves of *M. domestica *	100	26915	92664
	300	31052	70438
	1000	26967	107733
	1500	29317	123622
	2000	68229	105592
Vehicle-treated cell culture	(1 *μ*l)	34637	117913
