# Supplementary material for: Chemical Composition and In Vitro Cytotoxic Activity of Essential Oil of Leaves of Malus domestica Growing in Western Himalaya (India)
Source: Evid Based Complement Alternat Med. 2012 Apr 29;2012:649727. doi: 10.1155/2012/649727 (PMC3351209; doi:10.1155/2012/649727)
Supplement: Supplementary file 1 — The plant materials collected from 10 different populations of Chindi village of Mandi district on hydrodistillation procured 0.01% light pale colored oil on mfb with characteristic eucalyptol dominant flavour. All the samples were analyzed with the help of GC and GC-MS. All the samples qualitative GC pattern was found similar and the representative molecules concentration in the samples were as given below in Table 1A. [file 649727.f1.doc]

Table 1A: Chemical composition of *Malus domestica* essential oils from different samples of leaves from Chindi village of Mandi district, HP, India.

Compounds Retention index %

DB 5 A1 A2 A3

Eucalyptol 1031 43.7 41.0 34.1

Linalool 1095 1.0 0.9 0.9

4-Terpineol 1180 0.8 0.7 0.8

2-Undecanone 1288 1.1 1.0 1.5

β-Damascenone 1379 0.7 0.7 0.7

2-Dodecanone 1391 1.1 1.0 1.0

*trans*-Caryophyllene 1417 1.1 1.1 1.6

α-Farnesene 1506 9.6 9.1 11.1

1,6,10-Dodecatrien-3- 1542 2.0 1.9 2.2

ol-3,7,11-trimethyl

*cis*-3-Hexenyl benzoate 1567 3.1 3.1 4.7

*n*-Hexyl benzoate 1573 1.0 0.8 1.0

Benzyl benzoate 1762 1.7 1.5 1.6

6,10,14-Trimethyl-2-pentadecanone 1844 1.3 1.3 1.4

Podocarpene A 2036 3.8 3.4 3.7

Phytol 2112 11.5 10.8 8.3

Tricosane 2298 4.2 3.9 6.1

Pentacosane 2497 7.6 7.2 5.4
